# Prospective analysis of spatiotemporal variations in chill during winter, heat accumulation for flowering and spring frost in fruit trees in northeast Spain

**DOI:** 10.1007/s00484-026-03214-4

**Published:** 2026-05-01

**Authors:** Eduardo Pérez Sosa, Roberto Serrano-Notivoli, Helder Fraga, Miguel Ángel Saz, María Luz Hernández-Navarro

**Affiliations:** 1 Departamento de Geografía y Ordenación del Territorio, Instituto Universitario de Ciencias Ambientales (IUCA), Universidad de Zaragoza, Pedro Cerbuna 12, Zaragoza, 50009 España; 2 Centre for the Research and Technology of Agro-Environmental and Biological Sciences (CITAB), Institute for Innovation, Capacity Building, and Sustainability of Agri-Food Production (Inov4Agro), Department of Agronomy (Dagro), University of Trás-os-Montes e Alto Douro (UTAD), Quinta de Prados, Vila Real, 5000-801 Portugal

**Keywords:** Chill conditions, Dynamic model, Growing degree hours, Late frosts, Climate change, Fruit trees, Temperate climate, Thermal stretching

## Abstract

Agriculture is highly sensitive to climate variability, and ongoing warming is expected to modify the thermal conditions controlling fruit tree phenology and production. This study analyzes the spatiotemporal variability of winter chilling, spring heat accumulation, and spring frost probability across fruit-growing areas in Aragón (northeast Spain) using a regional, high-resolution agroclimatic approach. Chill portions (CP), growing degree hours (GDH), and spring frost probability occurrence (SFPO) were computed from daily gridded maximum and minimum temperature data at 1 km² spatial resolution. Agroclimatic indicators were derived using statistical, non-experimental methods based on established chilling and forcing models and empirical temperature thresholds. Recent historical variability was characterized using overlapping 30-year historical climate periods while future climate conditions were assessed using daily temperature projections from an ensemble of 18 regional climate models (EURO-CORDEX), dynamically downscaled from global climate models and bias-corrected. Projections were analyzed for a historical reference period (1971–2000) and under the intermediate and high emissions scenario RCP4.5 and RCP8.5 for mid-century (2041–2070) and late-century (2071–2100) periods. Climate projections at ~ 5 km spatial resolution were interpolated to 1 km and bias-corrected using Empirical Quantile Mapping with high-resolution observational data as reference. Changes in CP and GDH distributions were quantified using spatial differences and standardized anomalies relative to historical conditions, and indicators were extracted at the location of existing fruit orchards. Results indicate that effective winter chilling remains within broad ranges compatible with fruit production across all scenarios, with persistent spatial contrasts between western and eastern sectors. In contrast, spring heat accumulation shows a strong and spatially coherent increase, particularly in low-elevation and eastern areas, indicating an increasing influence of spring temperatures on phenological dynamics. Although future scenarios project a substantial reduction in the probability of frost occurrence after early March, increasing heat accumulation may advance phenological development, potentially shifting frost exposure to earlier periods in late winter. Overall, the results indicate that agroclimatic conditions in Aragón are strongly structured by regional climatic gradients, and that climate change is likely to intensify spatial contrasts between colder and warmer production areas rather than producing uniform changes across the region. These results provide a regional agroclimatic framework that can support adaptation planning and the long-term management of fruit production under climate change.

**Supplementary Information:**

The online version contains supplementary material available at 10.1007/s00484-026-03214-4.

## Introduction

The life cycle of temperate deciduous fruit trees is tightly controlled by environmental conditions, particularly photoperiod and temperature (Campoy et al. 2011). The progressive shortening of day length during autumn and winter, together with exposure to low temperatures, induces dormancy, a physiological state that inhibits growth and activates mechanisms of resistance to cold stress (Vilagrosa et al. 2006; Rohde and Bhalerao 2007; Lloret et al. 2018). Dormancy release requires the fulfilment of species- and cultivar-specific chilling requirements, after which heat accumulation governs the resumption of vegetative growth and reproductive development (Lloret et al. 2018; Ríos et al. 2018).

Under climate change, increasing temperatures at the end of winter and beginning of spring are expected to advance phenological development in many woody species (Richardson et al. 2013; Ma et al. 2019; Zohner et al. 2019; Chamberlain et al. 2021; Ettinger et al. 2021). This phenological advance may increase the exposure of sensitive developmental stages to late spring frost events, particularly in species with high phenological sensitivity, such as temperate deciduous fruit trees (Augspurger 2013; Ma et al. 2019). Importantly, frost damage does not depend solely on the occurrence of sub-zero temperatures, but on the coincidence between freezing events and vulnerable phenological stages, whose sensitivity varies among species and across developmental phases, with flowering and early fruit set being particularly critical in many fruit crops (Rodrigo 2000; Curzel and Hurtado 2020).

Spring frost risk therefore constitutes a compound process involving, on the one hand, the thermal conditions that regulate dormancy release and phenological advancement and, on the other hand, the probability that temperatures drop below critical thresholds after budburst (Rigby and Porporato 2008). Damage severity depends both on the minimum temperature reached and its duration as well as on the phenological stage affected, highlighting the need to jointly assess winter chilling, spring heat accumulation, and frost occurrence rather than analyzing these factors in isolation.

Previous studies have addressed different components of this problem using phenological models, critical temperature thresholds, or probabilistic approaches, often focusing on specific species or limited study areas. For example, Rigby and Porporato (2008) proposed a probabilistic framework combining degree-day models and frost occurrence, while Eccel et al. (2009) and Unterberger et al. (2018) integrated chilling and heat requirements with phenological observations to assess frost risk in apple-growing regions. More recently, Zahradníček et al. (2024) analyzed spring frost risk using the concept of “false spring” across multiple species and climatic thresholds. While these studies have provided valuable insights, their applicability at regional scales is often constrained by limited spatial coverage, the availability of long-term phenological series, or the use of climate projections with relatively coarse spatial resolution.

**Fig. 1:**
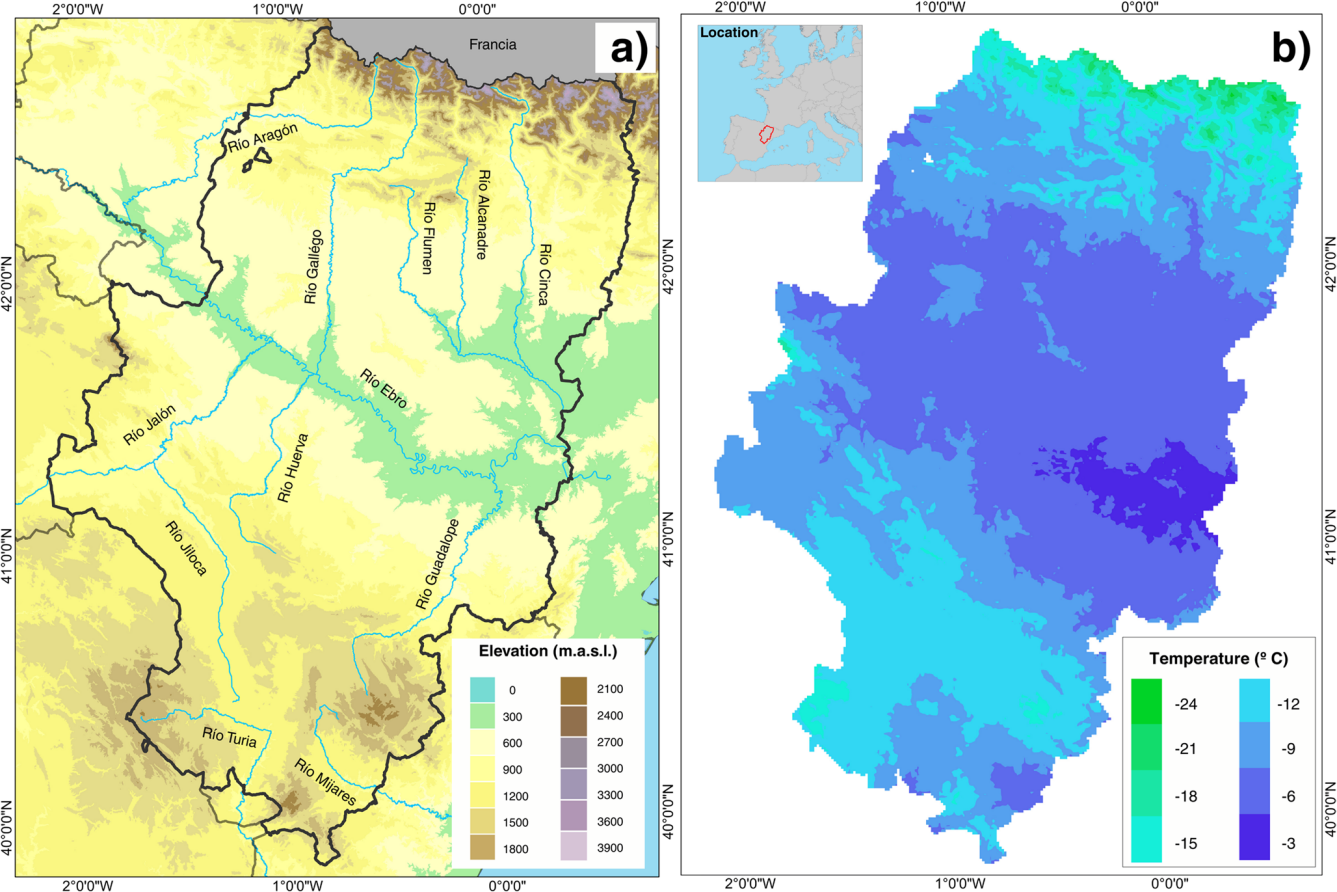
Elevation and minimum absolute temperature October-May 1950–2020 in Aragón. Aragón is located in the northeast of the Iberian Peninsula (**a**). Three main morphostructural forms are recognized: Pyrenees in the north (exceeding 3000 m.a.s.l.), Ebro depression in the central sector (80–300 m.a.s.l.) and the Iberian system in the south (almost reaching 2000 m.a.s.l.). The minimum absolute temperature ranges from − 3 °C in the central region to -24 °C in the high northern mountain areas (**b**)

These limitations are particularly relevant in regions characterized by strong climatic and topographic heterogeneity, such as Aragón, located in northeastern Spain (Fig. 1ab). Aragón is one of the main fruit-producing regions in the country and hosts a wide diversity of fruit species cultivated across contrasting environments, ranging from low-altitude continental basins to mountainous areas. The continental Mediterranean climate and complex orography favor the frequent occurrence of frost events, while future climate projections indicate substantial changes in temperature regimes that may alter both phenological timing and frost exposure (Hernández 1995; Cuadrat et al. 2007; Rodríguez et al. 2021; Egea et al. 2022).

At the national scale, several studies have evaluated chilling requirements of fruit trees in Spain under current and future conditions, including compilations of thermal requirements and analyses based on phenological records and climatic data (Fadón et al. 2020; Rodríguez et al. 2019, 2021). In Aragón, chilling estimates derived from phenological observations and climate data have been reported for selected species (Herrera et al. 2022; Fadón et al. 2023a, b, c), although their spatial representativeness is limited by the distribution of meteorological stations. In parallel, spatial indicators related to frost occurrence have been mapped for the Iberian Peninsula (Martínez-Núñez et al. 2015), and more recent studies have characterized frost variability and trends at regional and national scales (García-Martín et al. 2021; Egea et al. 2022; Sánchez et al. 2022). However, despite these advances, integrated assessments combining winter chilling, spring heat accumulation, and spring frost probability at high spatial resolution remain scarce at the regional scale.

In this context, the present study aims to analyze the spatiotemporal variability of winter chilling, spring heat accumulation, and spring frost probability at a regional scale in Aragón. Specifically, the objectives are to: (1) quantify thermal conditions at high spatial resolution using simulated climate scenarios representing past and future periods through chill portions (CP), growing degree hours (GDH), and spring frost probability occurrence (SFPO); (2) assess the spatial and temporal variability of these indicators across the territory; and (3) integrate chilling–heat (CP–GDH) and chilling–spring frost probability (CP–SFPO) relationships to identify the limits of fruit-growing areas and their potential shifts over time and space.

## Materials and methods

### Obtaining and processing data

#### Climate data

The daily maximum and minimum air temperatures were sourced from *SiCLIMA* (Serrano-Notivoli et al. 2024), an observational gridded climatic dataset at a high resolution (1 km^2^) for Aragón region that covers the period 1950–2020. It was built from the temperature records of more than 1200 weather stations through a comprehensive quality control, a data series reconstruction and a gridding process based on generalized linear mixed models (GLMMs) and generalized linear models (GLMs).

The high-resolution SiCLIMA dataset used in this study was previously validated against observed station data using a leave-one-out cross-validation approach. The validation showed very high correlations between observed and predicted values for both maximum and minimum temperatures (Pearson correlation > 0.97 and > 0.95, respectively), indicating a high reliability of the gridded temperature fields. However, as with any gridded dataset, the values represent estimates rather than direct observations, and uncertainties may be higher in mountain areas due to lower station density (Serrano-Notivoli et al. 2024).

#### Temperature under historical periods

Five overlapping 30-year periods were constructed (1951–1980, 1961–1990, 1971–2000, 1981–2010, and 1991–2020), shifting each window forward by one decade by removing the earliest 10 years and incorporating 10 new years. This moving-window approach ensures statistical robustness while enabling the assessment of temporal changes in mean climate conditions.

The 1971–2000 period was selected as the reference period, as it coincides with the calibration baseline used for bias adjustment of climate projections and represents the reference period of the climate model datasets. Accordingly, anomalies of mean CP and GDH values, as well as delta changes in SFPO, were computed relative to this period.

#### Temperature under climate scenarios

Daily maximum and minimum temperature projections were obtained from 18 climate models for a historical reference period (1971–2000), under the intermediate emissions scenario RCP4.5 and high emissions scenario RCP8.5 for mid-century period (2041–2070) and late-century (2071–2100) periods (Supplementary Table S1). Data were accessed through the THREDDS service of the AdapteCCa portal (https://escenarios.adaptecca.es/thredds/catalog/peninsula/Proyecciones_CMIP5_en_rejilla/catalog.html).

The climate projections originate from dynamically downscaled regional climate models (RCMs) developed within the EURO-CORDEX initiative, based on global climate model simulations (Hernanz et al. 2024). Original datasets at 0.11° spatial resolution were subsequently bias-adjusted and interpolated to 0.05° (~ 5 km) using the ISIMIP3 bias-correction framework, with the observational ROCIO_IBEB grid as reference for the historical period (Peral et al. 2017). The ISIMIP3 framework applies a trend-preserving bias adjustment based on quantile mapping techniques (Lange 2019; Lange and Büchner 2021).

##### Interpolation to 1 km and bias adjustment

To match the high spatial resolution of the SiCLIMA dataset, climate projections at 0.05° (~ 5 km) were first reprojected (EPSG:25830), and bilinearly interpolated to a 1 km grid and subsequently cropped and masked to the study area.

A multimodel ensemble was then constructed by computing the mean and standard deviation (sd) of the interpolated projections for the reference period 1971–2000 (hereafter referred to as AdapteCCa data). Corresponding daily values for the same period were extracted from the SiCLIMA dataset. To ensure temporal consistency, February 29 was removed from both historical and future datasets, resulting in time series of equal length (10,950 days).

Bias adjustment was subsequently applied at 1 km resolution using Empirical Quantile Mapping (EQM), in order to ensure statistical consistency between the interpolated projections and the SiCLIMA observations. EQM was implemented by computing empirical quantiles at a resolution of 0.05 between SiCLIMA and AdapteCCa data. In brief, the method involves: (i) estimating the empirical cumulative distribution function (CDF) of the observed data; (ii) estimating the CDF of the simulated data; (iii) determining the relative quantile of each simulated value; and (iv) mapping this quantile onto the observed distribution and applying the transfer function to future projections.

This procedure was calibrated over the 1971–2000 reference period and subsequently applied to future climate projections, implicitly assuming temporal stationarity of model bias, a common assumption in bias-correction approaches (e.g., Maurer et al. 2013).

As part of the diagnostic evaluation, the procedure was repeated using AdapteCCa data in place of future projections, generating an adjusted dataset (AdapteCCa-adjusted). Quantiles q5, q50, and q95 were then computed for SiCLIMA, AdapteCCa, and AdapteCCa-adjusted datasets, and quantile differences were calculated before and after adjustment (Supplementary Tables S2–S3; Supplementary Fig. S1).

#### Flowering dates and location of fruit tree areas

Flowering data for fruit trees were obtained from the Ministerio de Agricultura, Pesca y Alimentación (2024a, 2024b) and expressed as the percentage of trees in the flowering stage. The use of a standardized phenological observation scale could not be verified. Monthly flowering data were standardized and subjected to k-means clustering to classify fruit species according to their peak flowering period (Supplementary Table S4; Supplementary Fig. S2).

Fruit-growing areas were identified using the Sistema de Información Geográfica de Parcelas Agrícolas (SIGPAC). Spatial data corresponding to fruit tree plantations declared in 2024 were extracted for Aragón (Gobierno de Aragón, 2024). As plantation records do not specify species, plots classified as nut trees, fruit trees or mixed fruit systems were selected (Supplementary Fig. S3).

#### Thermal conditions and chill/forcing requirements

The main models for quantifying chilling requirements for dormancy and heat for flowering are the dynamic model and the growing degree hours model (Fishman et al. 1987a, b; Anderson et al. 1986). The idea of ​​the CP is based on the assumption that winter chill accumulates in two steps: (1) a chill intermediate product is formed from low temperatures, which can be destroyed by high temperatures; (2) when this intermediate product reaches a certain accumulation, it is permanently transformed into a chill portion (Fishman et al. 1987a, b; Luedeling et al. 2009; Fraga et al. 2019). This idea provides the dynamic model an advantage that allows for less variation in the quantification of chilling requirements in environments with warm conditions or warm winters. The advantages offered by the dynamic model have been stipulated in previous research (Luedeling et al. 2011; Salama et al. 2021), while heat accumulation for flowering represents the thermal integral required for flowering after breaking of dormancy (Ruiz et al. 2007). This accumulation takes place between the base threshold of 4 °C and the critical threshold of 36 °C, with an optimal temperature of 26 °C; when temperatures are higher than 36 °C or lower than 4 °C, the heat stops accumulating (Anderson et al. 1986).

Because hourly temperature data were not available, hourly temperatures were estimated from daily minimum and maximum temperatures using a diurnal temperature curve model parameterized by latitude and astronomical daylength, using the *chillR* package (Luedeling et al. 2024). The method assumes a sinusoidal temperature increase from sunrise to the daily maximum temperature and a logarithmic decrease from sunset to the following day’s minimum temperature, using temperatures from adjacent days to ensure a continuous nocturnal cooling curve. Sunrise, sunset and daylength were calculated as a function of latitude and day of year following Spencer (1971) and Almorox et al. (2005), allowing the reconstruction of a continuous hourly temperature series from daily Tmin and Tmax. This approach provides modeled rather than observed hourly temperatures and may introduce uncertainty because it assumes an idealized diurnal temperature pattern and does not account for short-term weather variability.

A fixed chilling period from November 1 to February 28 was adopted, consistent with the concept of “winter chilling” used in previous studies (Baldocchi and Wong 2008; Luedeling et al. 2009; Moral et al. 2021; Egea et al. 2022; Fernandez et al. 2023). Heat accumulation was calculated from February 1 to March 31, corresponding to the typical flowering period of most fruit species in the region (see Supplementary material section C. Flowering season of fruit trees in Aragón), in line with previous studies using similar or broader forcing windows (Egea et al. 2022; Fadón et al. 2023a, b, c). This period was selected to represent the main forcing phase preceding flowering for most species, rather than the exact flowering date, in order to provide a consistent regional indicator of spring heat accumulation.

Generalized threshold ranges of chilling and heat requirements were used to assess whether regional climatic conditions fall within ranges commonly associated with fruit production in Aragón. These ranges were derived from compilations of mean thermal requirements reported for major fruit tree species (Díez-Palet et al. 2019; Fadón et al. 2020; Delgado et al. 2021; Fadón Espiau et al. 2023) and encompass substantial intra- and inter- specific variability. This approach was intended to provide a regional climatic reference framework rather than species- or cultivar-specific suitability assessments.

#### Spring frosts probability of occurrence

Due to the lack of precise flowering dates, spring frost probability occurrence (SFPO, based on last frost date) was defined as the probability of minimum temperatures equal to or less than 0 °C occurring after March 1, a period during which most fruit species in the region are typically in bloom. Frost occurrence dates were computed as day-of-year values (1–365, covering the period from October to June).

Rather than assuming a theoretical distribution, frost occurrence dates were analyzed using the empirical cumulative distribution function (CDF). This non-parametric approach directly estimates the cumulative probability distribution from the observed data, avoiding any distributional assumptions. Consequently, the probability of frost occurrence after March 1 was calculated as one minus the CDF evaluated at day 60 (March 1), providing an empirical estimate of late frost risk for each climate scenario.

All agroclimatic indicators (CP, GDH, and SFPO) were calculated independently for each 1 km^2^ grid cell using daily temperature time series, and the resulting spatially explicit values were directly used to generate the maps.

### Analysis of temporal variability

The 2D scatter plots offer an alternative to traditional scatter plots when dealing with large datasets or when the goal is to highlight areas of higher data concentration. The resulting plot reveals where observations are most densely clustered within the most representative range of the data. Darker areas indicate higher density, meaning more frequent combinations of the two variables. Tighter density contours suggest a high concentration of data points, whereas broader contours indicate greater dispersion. The plot also provides insights into potential relationships between the variables. This representation was previously used by Fraga et al. (2019).

The 2D scatter plots were calculated, indicating the density and limits of CP-GDH and CP-SFPO values ​​for each pixel, based on filtered data (excluding values outside the range [q5, q95] for both variables) derived from the centroids of fruit crop areas. This filtering step was intended to minimize the influence of potential outliers. A subsequent step involved estimating the joint probability density of the observed data using a two-dimensional Kernel Density Estimation (KDE) with a gaussian kernel, generating values ranging between 0 and 1. As a non-parametric method, KDE allows for the approximation of the joint distribution without assuming a specific functional form. Density smoothing was controlled using bandwidths of 0.1 units. This approach is particularly useful for detecting patterns of data concentration in two dimensions.

Finally, three spatial indices were derived from standardized anomalies calculated relative to the 1971–2000 reference period. Magnitude was computed as the mean absolute anomaly to capture the overall intensity of change while avoiding cancellation effects between positive and negative values. Direction was defined as the mean anomaly, indicating the prevailing tendency of change within each grid cell. Variability was calculated as the standard deviation of anomalies, reflecting the dispersion of changes across periods and distinguishing consistent from heterogeneous responses.

## Results

### Spatial patterns of average CP, GDH and SFPO under climate scenarios in Aragón

**Fig. 2:**
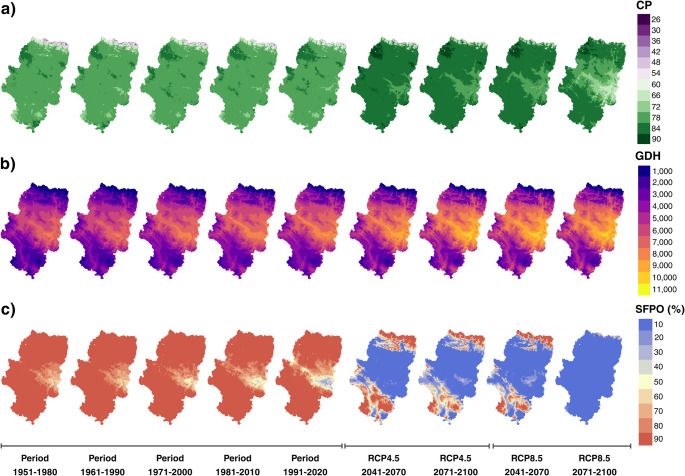
Patterns of average CP (**a**), GDH (**b**) and SFPO (**c**) according to historical periods and climate change scenarios in Aragón

In general, most of the Aragonese territory exhibited mean Chill Portion (CP) values within the ranges typically reported for winter chill in temperate fruit-growing regions (Fig. 2a). During the historical period, spatial patterns of CP were consistent, with much of the region maintaining values above 70 CP. Under both medium and high-emission scenarios for the medium and long term, an increase in effective CP was observed, except in the central zone, where CP values appear to decrease under the most extreme scenario.

Heat accumulation during February and March showed a pronounced elevational gradient, with higher GDH values at lower altitudes and lower accumulation at higher elevations (Fig. 2b). Although this spatial pattern remained constant across all scenarios, the magnitude of thermal forcing increased over time. Under climate change scenarios, pronounced warming was observed, particularly in the low-altitude central region, which became notably warmer than the surrounding territory.

Regarding the probability of frost after March 1st (SFPO), during the historical period, most of Aragón presented probabilities exceeding 80%, with slightly lower values concentrated in the central region. In contrast, the decline in SFPO across most of the territory (except for high-altitude areas) was highly conspicuous under future climate change scenarios, reaching considerably low levels in the most extreme scenario (Fig. 2c).

Overall, the spatial distribution of CP, GDH, and SFPO showed a clear regional structure in Aragon. Western and higher-altitude areas consistently exhibited greater chill accumulation and less heat accumulation, while eastern and lower-altitude areas showed less chill accumulation and greater heat accumulation. This spatial pattern remained constant in both historical and future scenarios, indicating that regional climate gradients associated with altitude and continentality are a dominant factor controlling agroclimatic conditions in the region.

### Anomalies CP, GDH and SFPO under climate scenarios in Aragón

Mean values and standard deviations of the differences (Δ) and standardized anomalies (Sa) of CP, GDH and SFPO for Aragón are presented in Supplementary E. Anomalies in Aragón .

**Fig. 3:**
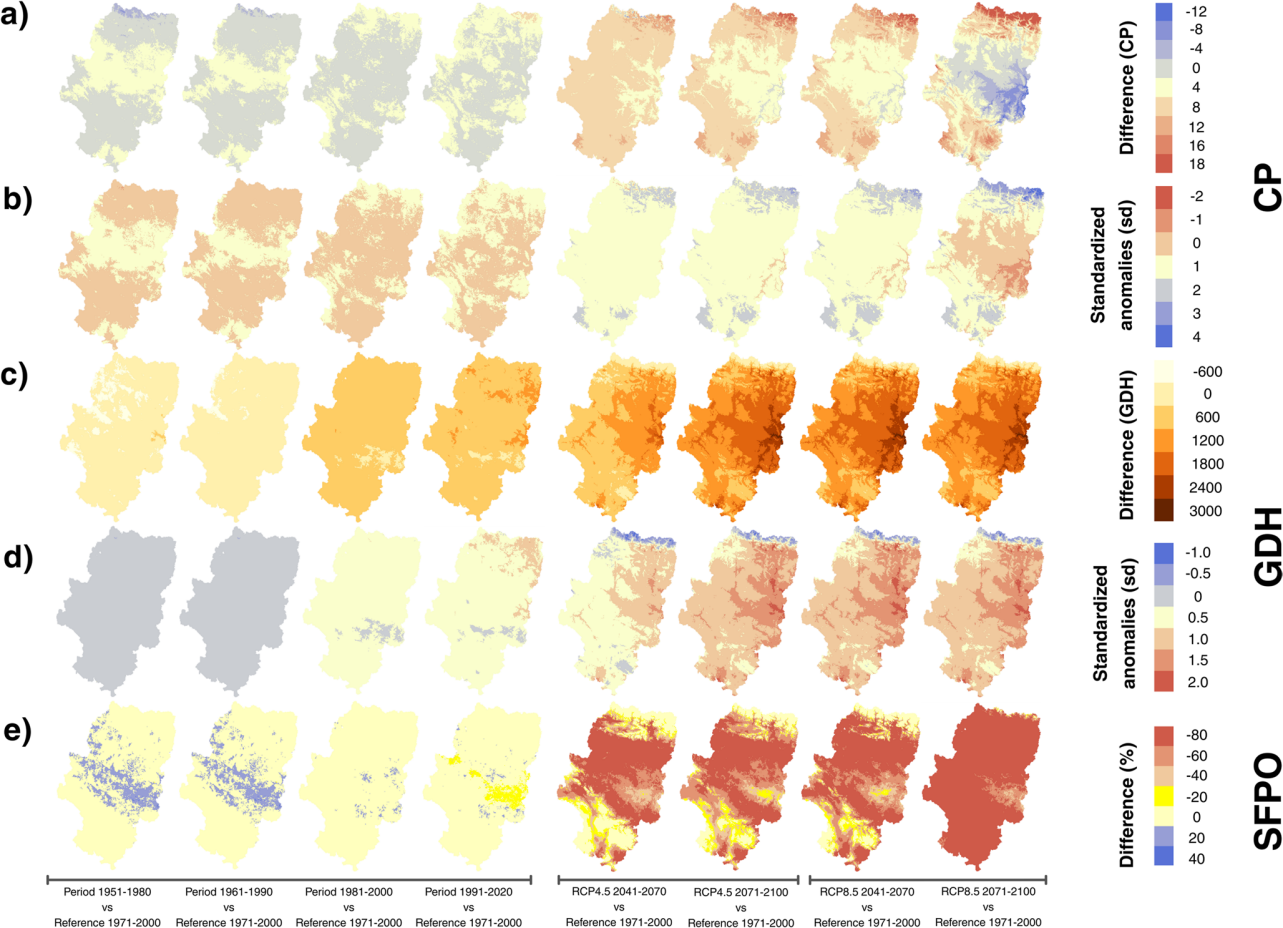
Patterns of differences and standardized anomalies of CP (**ab**), GDH (**cd**) and SFPO (**e**) according to historical periods and climate change scenarios in Aragón. The anomalies were calculated relative to the 1971–2000 reference period

Regional analysis reveals a profound transformation in the spatial distribution of CP and GDH, as well as the SFPO (Fig. 3).

During the historical period, variations in CP were moderate. The most significant discrepancies relative to the baseline period (1971–2000) were concentrated in the northern and southern fringes of the territory. In the 1951–1980, 1961–1990, and especially 1981–2010 periods, negative anomalies prevailed across approximately 70% of the territory (Supplementary Table S5). Under RCP4.5 climate scenarios, a generalized increase in chill efficiency is projected (mean anomaly of + 5.46 CP for 2041–2071), with greater availability in higher-altitude zones and a relative decrease in lowlands, primarily in the eastern sector. However, under the RCP8.5 scenario (2071–2100), a critical transition is observed: although the regional mean anomaly remains positive (+ 2.13), the spatial standard deviation rises to 6.41 (Supplementary Table S6). This reflects specific areas where CP decreases drastically (central-eastern sector), disrupting the trends of previous periods (Fig. 3ab).

Differences and standardized anomalies of GDH evidence an increasing thermal forcing throughout the Aragonese territory (Supplementary Table S7-S8). While the 1951–1980 period exhibited a mean negative anomaly of -449.2 GDH, future projections show an extreme acceleration, particularly in the eastern portion. By the end of the century (2071–2100), the RCP8.5 scenario projects mean increases of + 1049.3 GDH. In terms of standardized anomalies, this increase exceeds two standard deviations (Sa GDH > 0.80), consolidating a nearly universal spatial warming pattern across the region **(**Fig. 3cd).

The SFPO indicator shows remarkable historical stability relative to the baseline but undergoes a drastic shift in future projections (Supplementary Table S9). Under RCP4.5 scenarios, SFPO values decrease substantially across most of the territory, except for high-elevation areas. This trend reaches its peak in the RCP8.5 scenario for the 2071–2100 period, where the probability of frost after March 1 drops to a regional median of 13.0%. Spatially, this represents a reduction of up to 99.9% in the probability of late-season frost across a large part of the regional surface compared to the 1971–2000 period (Fig. 3e).

These results indicate that under the future scenarios, changes were not uniform across the territory, but rather that the intensity of existing regional climate gradients was modified. The spatial distribution of anomalies shows that eastern and lower-altitude areas experienced greater increases in heat accumulation and greater reductions in CP and SFPO.

### Analysis of the CP-GDH relationship under historical periods and climate change scenarios in fruit plantations in Aragón

The statistical summary of the CP-GDH relationship are presented in F. Statistical summary of CP-GDH and CP-SFPO in fruit trees plantations and the summary by region can be found in G. Statistical summary of CP, GDH and SFPO magnitude, direction and variability in east and west fruit trees areas in Aragón.

**Fig. 4:**
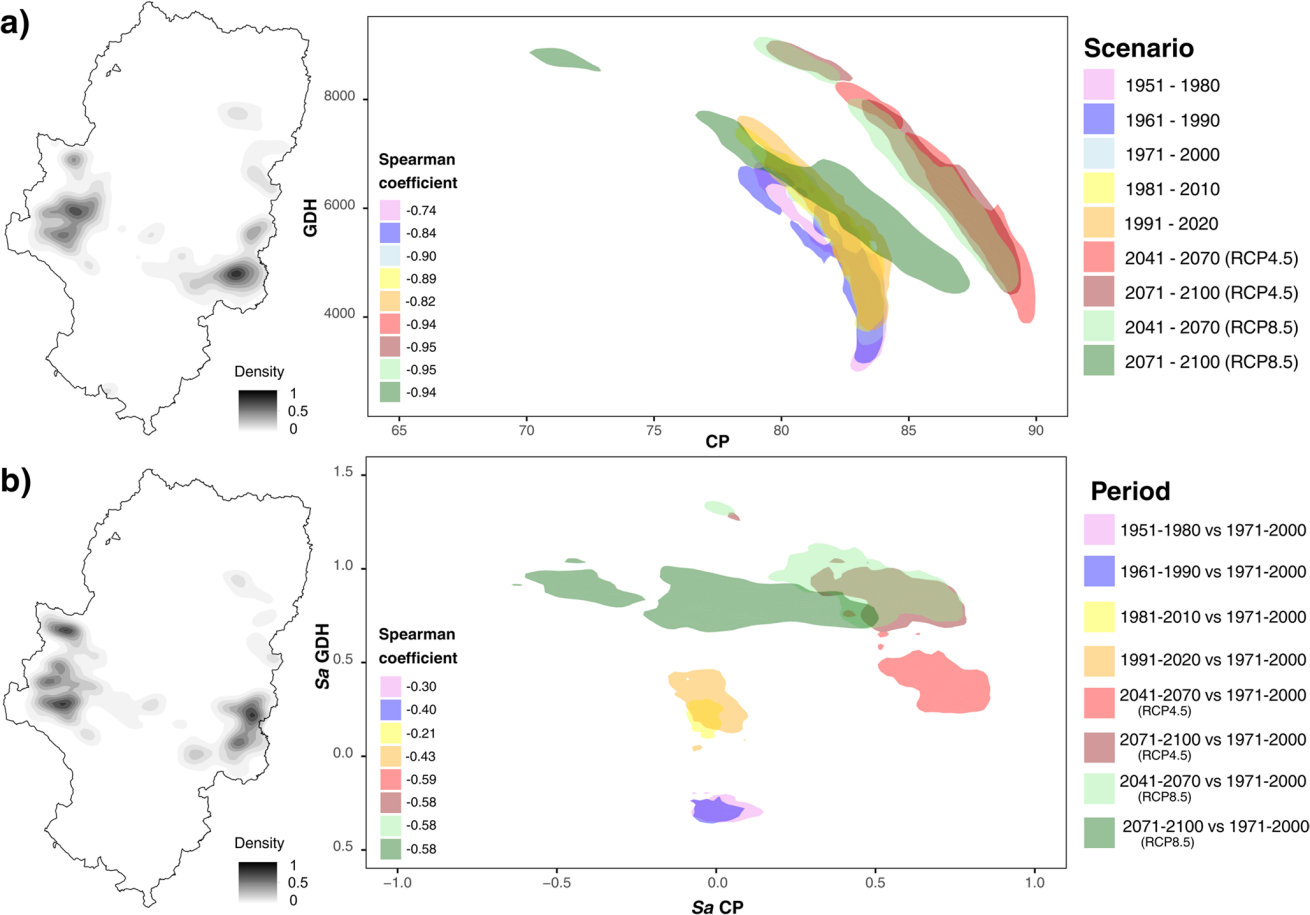
Spatial distribution and density patterns of CP-GDH (**a**) and standard anomalies (Sa) of CP-GDH (**b**) according to historical periods and climate change scenarios in fruit trees plantations in Aragón

The analysis of the areas with fruit tree presence showed a “thermal stretching” phenomenon, characterized by an increase in data dispersion that pushed the climatic conditions of some areas away from others, and a growing geographical polarization between the eastern and western sectors of Aragón (Fig. 4a**)**. The Interquartile Range (IQR) of CP in the cultivation areas quadrupled in the RCP8.5 scenario (2071–2100), increasing from a historical value of 2.1 to 7.9 (Supplementary Table S10). This spatial dispersion generated a significant gap: while 25% of the areas (upper quartile, q3) maintained levels of 84.1 CP, the most affected 25% (q1) dropped to 76.2 CP.

This behaviour indicates that agroclimatic conditions in Aragón are structured primarily by regional gradients rather than by uniform changes across the region. The progressive increase in dispersion reflects an increasing differentiation between colder western production areas and warmer eastern production areas.

Spearman correlation coefficients (rho) indicated a very strong inverse relationship in the spatial distribution of chilling and forcing, reaching a value of -0.9 (Supplementary Table S10). This value reflected that the map of production areas was divided in opposite ways: the sites that stood out for their high average CP were, due to their geographical location, those that recorded lower average forcing, and vice versa. The results confirmed that localities differentiated progressively according to their position in the region, concentrating cold conditions at one end of the map and heat at the other (Fig. 4a).

The summary of standardized anomalies in the fruit tree areas confirmed the magnitude of this shift (Supplementary Table S11). For the RCP8.5 scenario (2071–2100) compared to the reference period, the IQR of Sa CP stood at 0.78, starting from zero dispersion values in previous periods. In the case of forcing, Sa GDH showed an expansion of climatic extremes, with an IQR of 0.27 in the RCP8.5 scenario. This behavior reflected that fruit tree areas faced deviations from the historical average that were increasingly pronounced and heterogeneous (Fig. 4b).

**Fig. 5:**
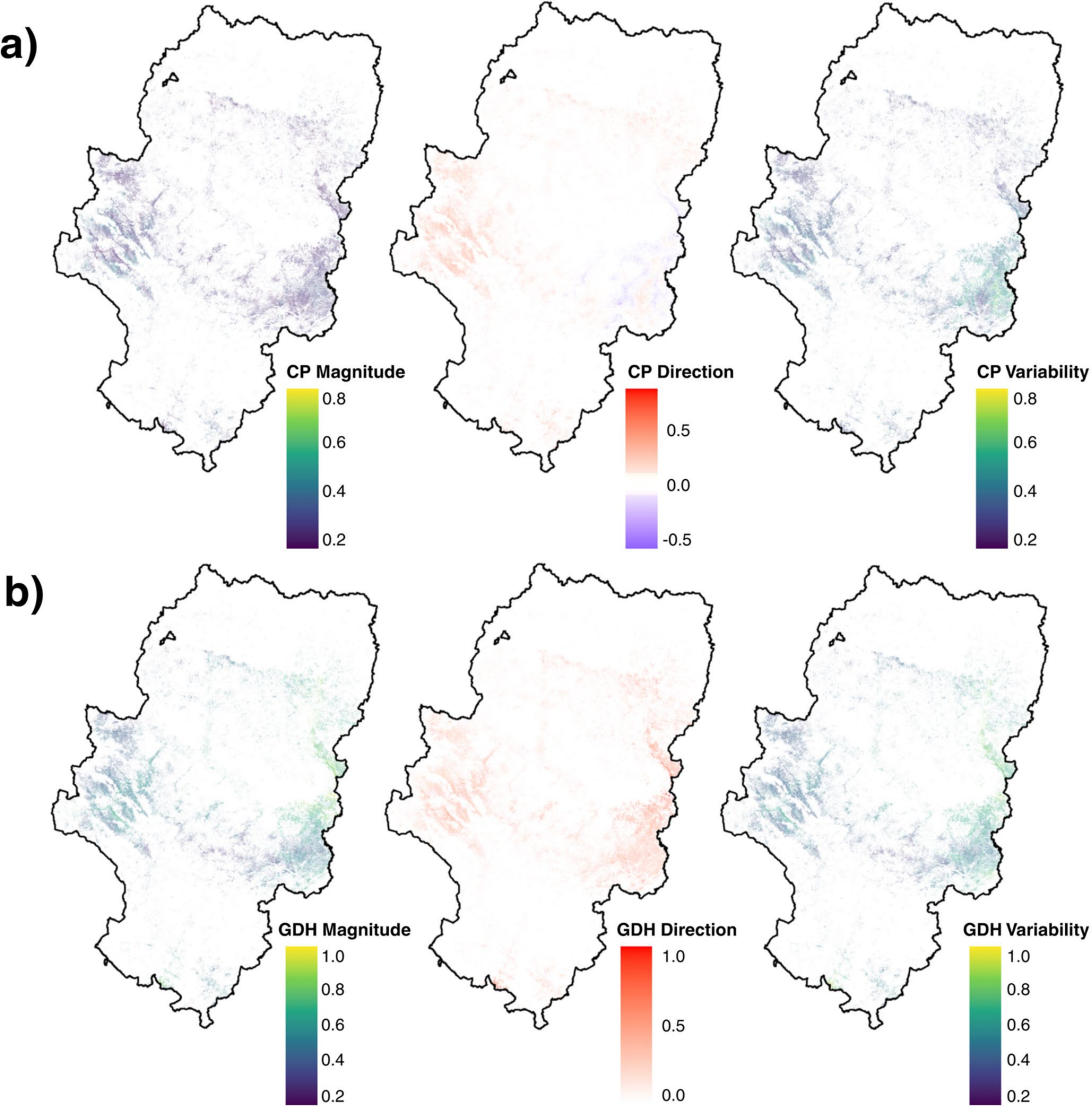
CP (**a**) and GDH (**b**) magnitude, direction and variability in fruit trees plantations in Aragón

To further explore this difference, the analysis of magnitude, direction, and variability indices confirmed that the East and West followed distinct paths. In the Western region, the shift toward higher CP were more intense, with a magnitude of 0.30 and a positive trend direction of 0.24. These changes in the West were slightly more stable over time, with a variability of 0.35. In contrast, the Eastern region showed an almost null direction of change for CP (0.02) and greater spatial heterogeneity (variability = 0.39) (Fig. 5a; Supplementary Table S14).

Regarding forcing, the Eastern region recorded the strongest changes in both magnitude (0.59) and the upward trend (direction = 0.46). Nevertheless, it was the zone with the most variable and least consistent results (variability = 0.58). The West showed a more moderate increase in heat (magnitude = 0.49 and direction = 0.34) and greater consistency in its evolution (variability = 0.48). Overall, the results indicated that the Western sector tended to consolidate as a more stable chill accumulation zone, while the Eastern sector was dominated by a more intense and variable increase in forcing (Fig. 5b; Supplementary Table S15).

### Analysis of the CP-SFPO relationship under historical periods and climate change scenarios in fruit plantations in Aragón

**Fig. 6:**
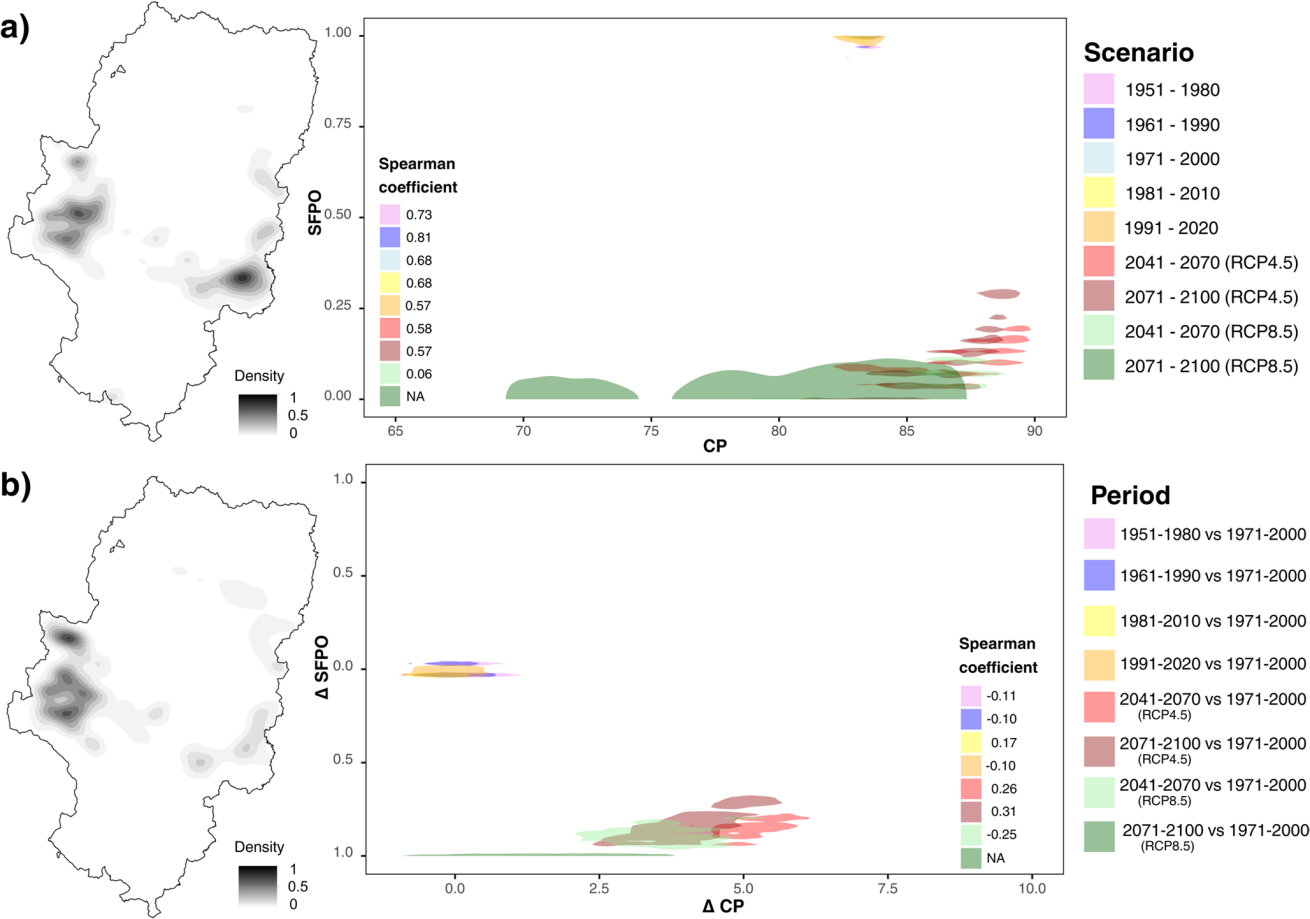
Spatial distribution and density patterns of CP-SFPO (**a**) and differences (Δ) of CP-SFPO (**b**) according to historical periods and climate change scenarios in fruit trees plantations in Aragón

The analysis of SFPO showed that the probability of occurrence of temperatures ≤ 0 °C after March 1st underwent a drastic decline across fruit trees areas. Historically, fruit tree areas presented a dispersion of 9.6%, with a median (q2) of 96.7%. However, under the RCP8.5 scenario (2071–2100), this indicator plummeted, with its median (q2) reaching 8.0% and its third quartile (q3) reaching 12.0%. This shift reflected that SFPO transitioned from a historical constant to an unlikely event across most of the territory, reducing its dispersion to an IQR of 7.0% (Fig. 6a; Supplementary Table S12).

Spearman correlation coefficients revealed a loss of structured association between CP and SFPO as the century progressed. While in the historical period the relationship was robust (rho = 0.7), indicating that colder areas had higher SFPO, this correlation decreased in future scenarios until becoming non-existent (NA) in the RCP8.5 scenario (Fig. 6a). This null value was due to SFPO falling to minimum levels.

The summary of SFPO differences in fruit tree areas confirmed the magnitude of this decline. For the RCP8.5 scenario (2071–2100) compared to the reference period, the differences showed a clear negative direction, with a q1 value of approximately − 70% and a median of around − 80%. The increase in heterogeneity within these differences, reflected in an IQR of about 13%, confirmed that the decrease in frost probability, although widespread, presented variations from the historical average among different fruit tree areas. Spearman correlation coefficients for the CP-SFPO differences were negative and very low (Fig. 6b; Supplementary Table S13).

**Fig. 7:**
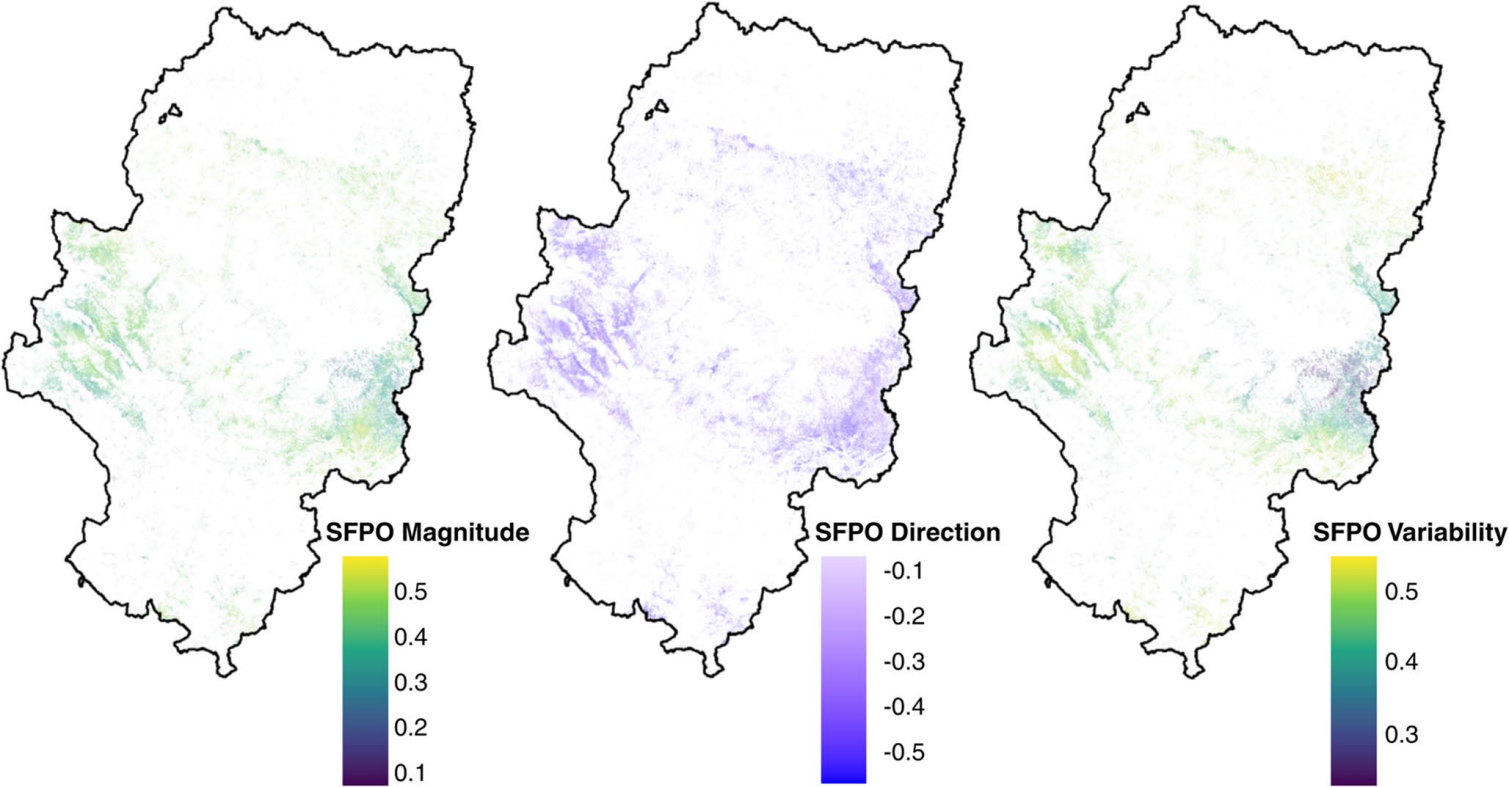
SFPO magnitude, direction and variability in fruit trees plantations in Aragón

Regarding magnitude, it was similar in both regions (around 0.4), and although both had a negative direction, it was slightly more pronounced in the west (-0.43). Likewise, it was in this same region where changes were more dispersed (variability of 0.45) compared to the east (0.40) (Supplementary Table S16). Overall, these results evidenced that the drastic reduction in frost risk, while massive throughout Aragón, maintained regional nuances in terms of the stability and depth of this climatic transition (Fig. 7).

The spatial patterns observed in the CP–SFPO relationship reinforce the existence of regional agroclimatic gradients, where colder areas historically presented higher frost probability and higher chilling accumulation, while warmer areas showed lower frost probability and higher heat accumulation. Under future scenarios, this regional structure remains, although frost probability decreases substantially across most areas.

Overall, the results indicate that agroclimatic conditions in Aragón are strongly structured by regional climatic gradients, particularly those associated with elevation and the west–east climatic transition. Climate change modifies these regional patterns by increasing heat accumulation, reducing frost probability, and increasing spatial contrasts between colder and warmer production areas. These results suggest that future agroclimatic changes are likely to be expressed primarily through regional climatic differences rather than through uniform changes across the territory.

## Discussion

We provide an integrated assessment of winter chilling, spring heat accumulation, and spring frost probability at high spatial resolution, enabling the identification of their spatiotemporal variability and their combined influence on fruit-growing areas in Aragón under past and future climate scenarios. The interpretation of these results should be framed within the use of generalized thermal thresholds, representing average ranges of chilling and heat requirements for the main fruit tree species cultivated in the region. These thresholds are not intended to assess the fulfilment of species- or cultivar-specific requirements, but rather to provide a regional reference framework to evaluate whether climatic conditions broadly fall within ranges compatible with fruit production.

### Winter chilling under climate change: efficiency rather than decline

Overall, winter chilling conditions in Aragón remain within the broad ranges of chilling requirements reported for most fruit tree species cultivated in the region across all analysed scenarios. Even under future climate scenarios, mean CP values remain above approximately 75 CP, indicating that, at the regional scale, no generalized loss of effective winter chilling is detected. This result is consistent with recent phenological studies indicating that current climate warming does not necessarily lead to a generalized shortage of winter chilling, although local effects may occur depending on species and climatic conditions (Jiang et al. 2025). This behaviour is consistent with recent studies showing that, in some Mediterranean and temperate regions, warming does not necessarily lead to a systematic reduction in accumulated winter chilling, particularly in environments where winters remain sufficiently cold (Fraga and Santos 2021; Freitas et al., 2023).

Nevertheless, winter chilling exhibits a well-defined spatial structure. The central and eastern sectors consistently show lower CP values than western and higher-elevation areas, although remaining within ranges compatible with the requirements of most fruit species cultivated in Aragón. In this context, spatial differences in chilling accumulation should be interpreted not as thermal deficits, but as regional contrasts that may modulate phenological responses under increasing spring warming. Similar patterns have been reported in studies comparing climatically contrasting sites, where genetically identical trees displayed divergent phenological behaviour driven by local climatic conditions (Fadón et al. 2023a, b, c).

### The dominant role of spring heat accumulation

In contrast to the relatively stable behaviour of winter chilling, spring heat accumulation shows a strong and spatially coherent increase across Aragón under future climate scenarios. This increase is progressive from historical to future scenarios, with particularly pronounced increases in low-elevation areas and in the eastern sector, indicating a clear intensification of thermal forcing during late winter and early spring. This pattern reinforces previous evidence indicating that warming during the forcing phase may exert a stronger influence on phenological timing than moderate changes in winter chilling, particularly in regions where chilling remains non-limiting (Fraga and Santos 2021; Freitas et al., 2023; Jiang et al. 2025).

The magnitude of the projected increase in GDH implies an acceleration of phenological development, likely leading to earlier flowering and fruit set. In regions where chilling requirements remain fulfilled, phenological timing is expected to become increasingly controlled by heat accumulation rather than by winter chilling. This behaviour is consistent with observed and projected trends towards earlier phenological phases in Europe and other temperate regions, where temperature increases during the forcing period have been identified as a primary driver of phenological advancement at the global scale (Campoy et al. 2011; Piao et al. 2019; Zohner et al. 2019).

From an agroclimatic perspective, these results suggest that future changes in fruit production systems in Aragón will be more strongly associated with increasing heat accumulation and phenological advancement than with insufficient winter chilling.

### Spatial decoupling between chilling and heat accumulation

The negative spatial relationship between CP and GDH strengthens under climate change scenarios, indicating an increasing spatial differentiation between areas characterized by high winter chilling accumulation and those characterized by high spring heat accumulation. This relationship does not represent a physiological chilling–forcing relationship, but rather the spatial organization of agroclimatic conditions across Aragón, where colder and higher-elevation areas accumulate more winter chill but less spring heat, while warmer and lower-elevation areas accumulate less chill but more heat.

Under future climate scenarios, this spatial contrast becomes more pronounced, as heat accumulation increases across the entire region while chilling accumulation shows more heterogeneous spatial behaviour. As a result, some areas remain predominantly chill-dominated, while others become increasingly heat-dominated. This pattern reflects the increasing influence of regional climatic gradients, particularly those associated with elevation and the west–east climatic transition.

This behaviour has important agroclimatic implications. In western Aragón, where chilling accumulation is higher, increased heat may induce earlier flowering without compromising dormancy release. In eastern areas, where chilling accumulation is systematically lower but generally remains within suitable ranges, the increase in thermal forcing becomes the dominant driver of phenological dynamics. In these environments, the primary source of change is not necessarily a lack of chilling, but the increasing importance of spring heat accumulation and its interaction with phenological timing.

This pattern is consistent with previous evidence showing that climate warming can alter the balance between winter chilling and spring heat accumulation in fruit tree systems (Luedeling et al. 2009; Fraga and Santos 2021). In particular, moderate winter warming may increase effective chilling when temperatures shift from sub-zero values to chilling-effective ranges, while simultaneously enhancing heat accumulation during the forcing period, as observed in other warming regions (Guo et al. 2014). Under such conditions, phenological responses may become increasingly driven by spring heat accumulation rather than by chilling limitation, even when winter chilling requirements remain fulfilled. Chilling and forcing act sequentially and jointly to regulate budburst and flowering, and their relative influence may vary depending on local climatic conditions and temperature regimes (Hao et al. 2024).

### Spring frost risk under advancing phenology

Although future scenarios indicate a substantial reduction in the probability of frost occurrence after March 1, this decrease does not necessarily translate into a proportional reduction in production risk. The increase in spring heat accumulation observed across the region is likely to advance phenological development, potentially shifting sensitive developmental stages to earlier dates and maintaining exposure to infrequent but potentially damaging frost events.

Under this scenario, the frost events that may pose the greatest risk to fruit production may no longer be limited to those occurring after March, which would represent rare events, but rather those occurring in late winter, particularly in February, when advancing phenology may place sensitive stages earlier in the season. Therefore, frost risk should be interpreted as a shift in exposure timing rather than simply as a reduction in frost frequency.

This behaviour is consistent with conceptual frameworks suggesting that frost risk may remain unchanged or even increase when phenological advancement outpaces the extension of frost-free periods (Augspurger 2013; Vitasse et al. 2018; Ma et al. 2019). Observational studies across the Iberian Peninsula have also reported decreasing frost frequency alongside persistent episodic frost damage, underscoring the complexity of frost risk under climate change (García-Martín et al. 2021; Sánchez et al. 2022).

From a regional agroclimatic perspective, these results suggest that the main change is not only a reduction in frost probability, but a shift in the interaction between frost occurrence and phenological timing, increasingly influenced by heat accumulation. Accordingly, changes in frost probability should be interpreted as shifts in exposure patterns rather than as precise projections of extreme frost occurrence at specific locations.

### Thermal stretching and increasing agroclimatic heterogeneity

One of the most relevant spatial results of this study is the increase in the dispersion of agroclimatic conditions across fruit-growing areas, particularly under the high-emission scenario. The increase in the interquartile range of CP and GDH indicates a progressive expansion of the agroclimatic space within the region, a process that can be described as thermal stretching.

Rather than a uniform shift in climatic conditions across Aragón, the results indicate an increasing divergence between colder and warmer production areas. Western and higher-elevation areas tend to maintain higher chilling accumulation and more moderate heat accumulation, while eastern and lower-elevation areas show lower chilling and stronger increases in heat accumulation.

This increasing spatial heterogeneity suggests that climate change may lead to an internal reorganization of fruit-growing areas within the region rather than a simple regional loss of suitability. Spatial variability in chilling and forcing requirements across climatic gradients has been reported in temperate tree species, reflecting the influence of local temperature regimes on dormancy release and flowering timing (Hao et al. 2024).

Taken together, the results suggest that the spatial distribution of fruit-growing areas in Aragón may remain relatively stable in the short to medium term within the agroclimatic ranges considered in this study, although gradual shifts are expected due to increasing spring temperatures and the growing spatial differentiation between chilling-dominated and heat-dominated areas. In this context, warmer spring conditions may increasingly favour late-flowering species or cultivars, whereas early-flowering species may face greater interannual variability in frost exposure due to earlier phenological development and the persistence of late winter frost events.

This interpretation is consistent with previous studies documenting heterogeneous phenological responses across species and regions. For example, delayed or absent advances in flowering have been reported for some early-flowering species despite warming trends (Martínez-Lüscher et al. 2017; Delgado et al. 2021), while adverse effects associated with warm winters and chilling-related constraints have been described in Mediterranean environments (Bartolini et al. 2019), as well as in other temperate regions experiencing winter warming (Guo et al. 2014). Conversely, recent evidence suggests that later-flowering species or populations located in cooler environments may be less vulnerable to false spring events (Bosdijk et al. 2024).

### Potential agronomic implications for fruit species

The agroclimatic ranges identified in this study are consistent with reported chilling and heat requirements for several fruit species cultivated in northeastern Spain. For example, apricot, cherry, plum, peach, and pear typically accumulate between approximately 30 and 85 chill portions and between 3000 and 9000 growing degree hours depending on species and cultivar (Herrera et al. 2022; Fadón et al. 2023a, b, c; Guerrero et al. 2024; Drogoudi et al. 2023; Fadón Espiau et al., 2023). Similar ranges have been reported for apple cultivars in other Spanish regions, with chilling between approximately 35 and 88 CP and heat accumulation between 6000 and 16,000 GDH (Díez-Palet et al. 2019; Delgado et al. 2021).

These ranges overlap with the agroclimatic conditions observed across Aragón under both historical and future scenarios, indicating that climate change does not necessarily imply a generalized loss of climatic suitability for fruit production. Instead, the main changes are related to increasing heat accumulation, reduced frost probability, and increasing spatial heterogeneity. From a phenological perspective, flowering in temperate fruit trees is regulated by the sequential accumulation of winter chilling and spring heat, with chilling controlling dormancy release and heat accumulation determining the rate of bud development and flowering timing. When chilling requirements are fulfilled, flowering phenology becomes primarily controlled by heat accumulation, which explains the strong influence of spring temperatures observed in this study (Hao et al. 2024; Jiang et al. 2025).

According to the flowering periods compiled in the Supplementary Material C. Flowering season of fruit trees in Aragón, almond typically flowers between February and March, apricot and cherry mainly in March, peach, plum, nectarine and pear in April, and apple and quince mostly in April–May. Although the use of a standardized phenological observation scale could not be verified for all sources, these flowering periods provide a reasonable approximation of the typical blooming windows for the main fruit species in the region. When these flowering periods are considered together with the projected increase in heat accumulation and the reduction in frost probability after March 1, the results suggest that early-flowering species may continue to be exposed to frost events despite the overall decrease in frost probability, whereas later-flowering species may experience reduced frost exposure but significant phenological shifts driven by increasing heat accumulation.

### Methodological strengths and uncertainty

A key strength of this study lies in the generation of climate fields at a 1 km² spatial resolution, which allowed the identification of fine-scale agroclimatic gradients that would not be detectable using coarser datasets. As with any statistical downscaling approach, the resulting fine-scale spatial patterns reflect both the underlying climate signal and the methodological choices involved in the downscaling process (Wilby et al. 2004); therefore, the robustness of the results lies primarily in the relative spatial contrasts and temporal changes rather than in absolute point-scale values.

In this context, the reliability of the agroclimatic indicators presented in this study depends largely on the accuracy of the high-resolution temperature fields, which have been previously validated against station observations (Serrano-Notivoli et al. 2024).

The bias correction procedure applied to temperature variables further increases confidence in the results. EQM substantially reduced systematic biases in both minimum and maximum temperatures, particularly across central and upper quantiles, improving the estimation of heat accumulation and conditions relevant for chilling and frost occurrence. Nevertheless, local discrepancies persist in the lower tail of the minimum temperature distribution, likely associated with strong topographic heterogeneity and the inherent limitations of statistical bias correction methods in reproducing rare events at local scales. Accordingly, while the identified spatial and temporal trends are robust, point-scale estimates of rare frost events should be interpreted with caution.

An additional source of uncertainty is related to the assumption of bias stationarity inherent in most bias correction approaches. In this study, bias correction was calibrated using the historical period (1971–2000) and applied to future climate projections, which implicitly assumes that model biases remain constant over time. While this assumption is commonly adopted in climate impact studies, it may not always hold under changing climate conditions (Maurer et al. 2013). Therefore, the projected values should be interpreted primarily in terms of relative changes and spatial patterns rather than as exact absolute projections of future temperature conditions. This consideration is particularly relevant for this study, as the main objective is to identify regional agroclimatic patterns and relative changes rather than precise local-scale projections.

The use of reconstructed hourly temperatures introduces some uncertainty because the method assumes an idealized diurnal temperature cycle and does not account for short-term meteorological variability such as cloud cover, wind, or rainfall. Therefore, deviations between reconstructed and actual hourly temperatures may occur, particularly during days with rapid temperature changes, which may affect the estimation of temperature-based hourly metrics. However, previous studies have shown that reconstructed hourly temperature series reproduce seasonal thermal accumulation with reasonable accuracy, particularly when the objective is to analyze regional patterns rather than exact phenological dates (Luedeling et al. 2009). Therefore, this uncertainty is unlikely to substantially affect the main regional patterns and trends identified in this study.

### Limitations and future research

The assessment of thermal sufficiency was based on generalized ranges of chilling and heat requirements, derived from compilations of average requirements of major fruit tree species and encompassing wide intra- and interspecific variability. Consequently, the results do not identify species- or cultivar-specific limitations, but rather highlight the regional dynamics of interactions between chilling, heat accumulation, and frost risk under climate change scenarios.

Similarly, in the absence of precise flowering dates, spring frost probability was defined as the probability of minimum temperatures equal to or below 0 °C occurring after 1 March, a period during which most fruit species in the region are typically in bloom. While this approach does not capture species- or cultivar-specific phenological sensitivity, it provides a consistent regional proxy for frost exposure under past and future climate scenarios.

Future research should integrate long-term phenological observations linked to specific species and cultivars in order to refine agroclimatic thresholds, improve phenological modelling, and better quantify the interaction between phenological shifts and frost risk under climate change.

## Conclusions

This study provides a high-resolution agroclimatic assessment of winter chilling, spring heat accumulation, and spring frost probability across Aragón under past and future climate scenarios, allowing the analysis of their spatial variability and combined influence on fruit-growing areas.

The results show that, at the regional scale, effective winter chilling remains within ranges broadly compatible with the chilling requirements of most fruit tree species cultivated in the region, with no evidence of a generalized loss of winter chilling. Nevertheless, persistent spatial contrasts associated with altitudinal and regional climatic gradients play a key role in structuring agroclimatic conditions across Aragón and may modulate phenological responses under warming conditions.

In contrast, spring heat accumulation exhibits a strong and robust increasing signal, particularly in low-elevation areas and in the eastern sector of Aragón. This increase in thermal forcing suggests a growing influence of spring temperature conditions in shaping phenological dynamics, in a context where winter chilling remains broadly sufficient at the regional scale.

Although future scenarios indicate a substantial reduction in the probability of frost occurrence after early March, this decrease does not necessarily translate into a proportional reduction in production risk. The projected increase in heat accumulation may advance phenological development, potentially shifting frost exposure to earlier periods in late winter. Therefore, changes in frost probability should be interpreted as shifts in exposure timing rather than simply as a reduction in frost frequency.

Taken together, the results indicate that climate change in Aragón is likely to be expressed not as a uniform agroclimatic shift, but as an increasing spatial differentiation between colder and warmer production areas, associated with changes in heat accumulation, frost exposure timing, and regional climatic gradients. Under this scenario, major fruit-growing areas may remain relatively stable in the short to medium term, although increasing spring temperatures and changing frost exposure may differentially affect species depending on flowering timing and regional climatic conditions.

This study highlights the value of high-resolution climate information for identifying regional agroclimatic patterns and supporting adaptation strategies. Future research should integrate long-term phenological observations and species- or cultivar-specific requirements in order to refine agroclimatic assessments and improve the evaluation of frost risk under climate change.

## Supplementary Information

Below is the link to the electronic supplementary material.

Supplementary Material 1 (PDF 847 KB)

## Data Availability

The datasets are available in Repository name: Zenodo Data identification number: 10.5281/zenodo.12822293.
