# Supplementary material for: Prospective analysis of spatiotemporal variations in chill during winter, heat accumulation for flowering and spring frost in fruit trees in northeast Spain
Source: Int J Biometeorol. 2026 May 1;70(5):151. doi: 10.1007/s00484-026-03214-4 (PMC13134984; doi:10.1007/s00484-026-03214-4)
Supplement: Supplementary file 1 — Supplementary Material 1 (PDF 847 KB) [file 484_2026_3214_MOESM1_ESM.pdf]

Supplementary materials for 'Prospective analysis of spatiotemporal variations in chill during winter, heat accumulation for flowering and spring frost in fruit trees in northeast Spain'

Eduardo Pérez Sosa[1], Roberto Serrano-Notivoli[1], Helder Fraga[2], Miguel Ángel Saz[1], María Luz Hernández-Navarro[1]

eduardo.perez@unizar.es, roberto.serrano@unizar.es, hfraga@utad.pt, masaz@unizar.es, mlhernan@unizar.es

Departamento de Geografía y Ordenación del Territorio. Universidad de Zaragoza. Pedro Cerbuna 12, 50009, Zaragoza, España

[1] Departamento de Geografía y Ordenación del Territorio. Instituto Universitario de Ciencias Ambientales (IUCA). Universidad de Zaragoza. Pedro Cerbuna 12, 50009, Zaragoza, España.

[2] Centre for the Research and Technology of Agro-Environmental and Biological Sciences (CITAB), Institute for Innovation, Capacity Building, and Sustainability of Agri-Food Production (Inov4Agro), Department of Agronomy (Dagro), University of Trás-os-Montes e Alto Douro (UTAD). Quinta de Prados, Edifício Reitoria Room D2.30, 5000-801 Vila Real, Portugal.

Corresponding authors.

e-mail addresses: eduardo.perez@unizar.es ; 848159@unizar.es (EPS)

Introduction

Climate change and global warming pose major threats to the successful cultivation of these species. Aragon is home to some of the nation's main fruit-producing regions. Therefore, it is necessary to analyze the variability of the climate resource in the region and its future prospects for agricultural planning and land use planning. In this document, we provide supplementary materials for the work 'Prospective analysis of spatiotemporal variations in chill during winter, heat accumulation for flowering and spring frost in fruit trees in northeast Spain' developed by Pérez et al. (2026).

A. CMIP5 Climate Models

According to Hernanz et al. (2024), these models are part of the EURO-CORDEX initiative based on the global CMIP5 simulations, which currently provides projections using various regional climate models (RCMs), nested within different global climate models from CMIP5, for the historical scenario and the RCP4.5 and RCP8.5 emissions scenarios (**Supplementary Table S1**). This dataset has been interpolated from its original resolution (0.11°) to a resolution of 0.05° (5 km), using a bias correction procedure based on the ISIMIP3 method (Lange, 2019; Lange & Büchner, 2021).

Supplementary Table S1. Grid projections from dynamic regionalization's (RCMs), generated in the EURO-CORDEX initiative from CMIP5 global simulations.

**Global model**	**Regional model**
CNRM-CERFACS-CNRM-CM5	CLMcom-CCLM4-8-17
CNRM-CERFACS-CNRM-CM5	CNRM-ALADIN63
CNRM-CERFACS-CNRM-CM5	KNMI-RACMO22E
CNRM-CERFACS-CNRM-CM5	SMHI-RCA4
ICHEC-EC-EARTH	CLMcom-CCLM4-8-17
ICHEC-EC-EARTH	DMI-HIRHAM5
ICHEC-EC-EARTH	KNMI-RACMO22E
ICHEC-EC-EARTH	SMHI-RCA4
IPSL-IPSL-CM5A-MR	IPSL-WRF381P
IPSL-IPSL-CM5A-MR	SMHI-RCA4
MOHC-HadGEM2-ES	CLMcom-CCLM4-8-17
MOHC-HadGEM2-ES	DMI-HIRHAM5
MOHC-HadGEM2-ES	KNMI-RACMO22E
MOHC-HadGEM2-ES	SMHI-RCA4
MPI-M-MPI-ESM-LR	CLMcom-CCLM4-8-17
MPI-M-MPI-ESM-LR	MPI-CSC-REMO2009
NCC-NorESM1-M	DMI-HIRHAM5
NCC-NorESM1-M	GERICS-REMO2015
NCC-NorESM1-M	SMHI-RCA4

Source: AdapteCCa (https://escenarios.adaptecca.es/thredds/catalog/peninsula/Proyecciones_CMIP5_en_rejilla/catalog.html).

B. Bias correction for minimum and maximum temperatures

Supplementary Tables S2-S3 and **supplementary Figure S1** show that, prior to bias correction, minimum temperatures (Tmin) exhibited systematic negative biases, particularly at the median and upper quantiles (q50 and q90), with values reaching up to −4.6 °C and a high degree of spatial dispersion. After applying Empirical Quantile Mapping (EQM), median and mean biases for these quantiles were reduced to values close to zero, indicating an effective correction of systematic bias in the central and upper parts of the Tmin distribution, which is critical for the estimation of winter chilling (in CP units) and spring frost probability occurrence (SFPO). Nevertheless, local extreme values persisted at the lower quantile (q5), likely associated with topographic complexity and the inherent limitations of statistical bias correction methods in reproducing rare temperature extremes at high spatial resolution.

For maximum temperatures (Tmax), pre-adjustment biases were even more pronounced, reaching values close to −9 °C at the upper quantile. Following EQM adjustment, biases were effectively removed across all evaluated quantiles (q5, q50, and q90), with median and mean values centered around zero and a very narrow interquartile range (approximately ±0.2 °C). This strong performance confirms the robustness of heat accumulation estimates and demonstrates that the spatial patterns and temporal trends observed for chill portions, forcing and spring frost probability occurrence are not artifacts of temperature bias in the climate data, although estimates of rare extreme events should be interpreted in terms of regional tendencies rather than exact point-scale values.

Supplementary Table S2. Quantile bias before and after adjustment for minimum temperatures.

Quantile	Min	Q1	Median	Mean	Q3	Max
q5 before	-0.29	2.25	2.76	2.80	3.27	8.62
q5 after	0.10	2.29	3.50	3.88	5.15	15.89
q50 before	-3.83	-0.51	-0.06	-0.04	0.37	4.74
q50 after	-0.14	-0.02	-0.01	0.05	0.03	6.19
q90 before	-4.56	-1.11	-0.65	-0.62	-0.19	4.47
q90 after	-0.19	-0.14	0.02	0.02	0.05	0.23

Supplementary Table S3. Quantile bias before and after adjustment for maximum temperatures.

Quantile	Min	Q1	Median	Mean	Q3	Max
q5 before	-1.01	2.09	2.66	2.83	3.33	10.68
q5 after	-0,30	-0.05	-0.01	0.11	0.03	9.30
q50 before	-6.96	-0.19	0.18	0.25	0.63	7.33
q50 after	-0,18	-0.03	-0.01	-0.01	0.02	0.17
q90 before	-9.11	-1.35	-0.74	-0.63	0.07	5.50
q90 after	-0.16	-0.01	-0.01	0.01	-0.01	0.26

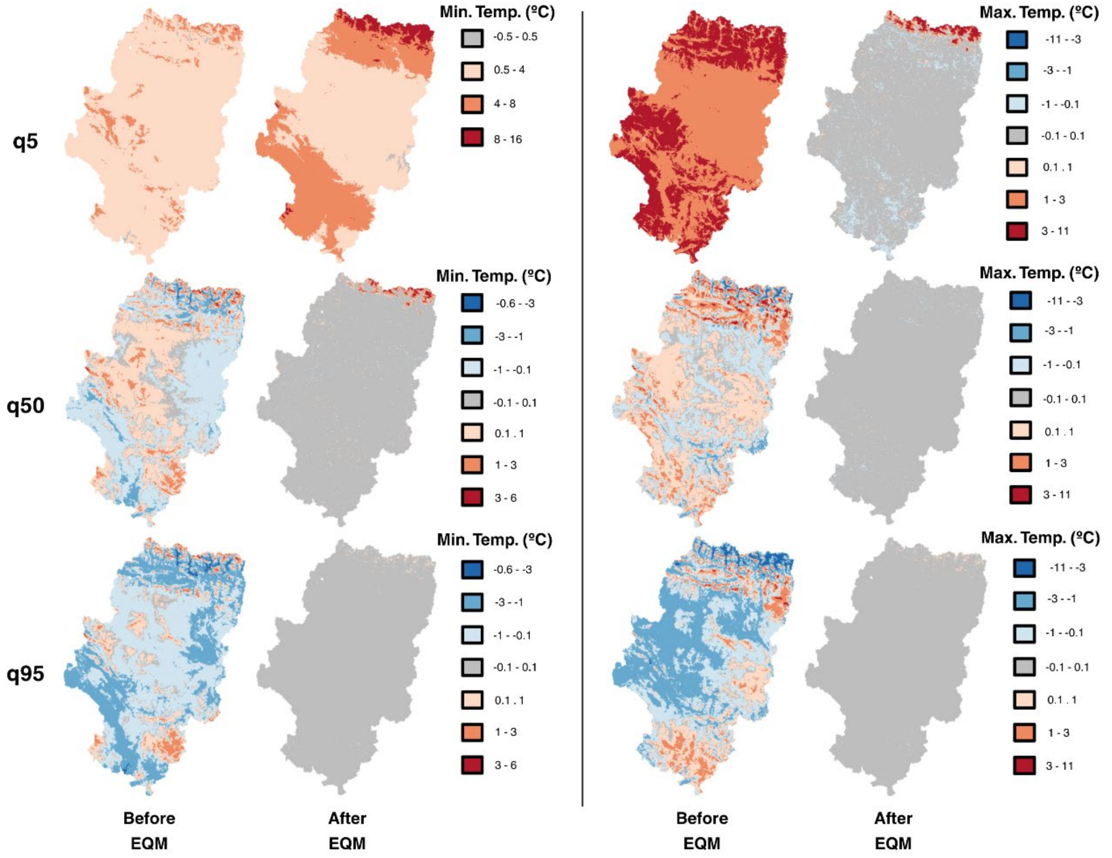

Supplementary Figure S1. Quantile bias before and after adjustment for minimum and maximum temperatures for daily data SiCLIMA and ensemble models from AdapteCCa (period 1971-2000).

C. Flowering season of fruit trees in Aragon

Supplementary Table S4. Months in which the highest flowering peaks occur for different fruit trees in Aragon.

Fruit trees	Month	Flowering (%)
Hazel tree	March	100
Apricot tree	March	82
Walnut	March	80
Cherry and sour cherry	March	70
Almond tree	March	60
Pear var. Limonera	April	70
Pear var. Ercolini	April	62
Pear var. Conferencia	April	60
Peac htree	April	53
Plum tree	April	53
Nectarine	April	50
Pear var. Blanquilla	April	43
Quince tree	May	90
Apple tree var. Fuji	May	45
Apple tree var. Gala	May	43
Other pear trees	May	40
Apple tree var. Starking	May	30
Other apple trees	May	30

Source: Ministerio de Agricultura, Pesca y Alimentación (2024a, 2024b).

Supplementary Figure S2 shows the grouping of fruit trees in Aragon based on the percentage of flowering per hectare according to the Ministerio de Agricultura, Pesca y Alimentación (2024a, 2024b). Group 1 consists of whose flowering is concentrated mainly during the months of March (average >83% ±13%) and April (average 17% ±13%). Apricot trees, hazelnut trees, cherry and sour cherry trees, and walnut trees belong to this group. Group 2 consists of spring-flowering fruit trees (March-May), with a predominance in April (average 56% ± 9%). Peach and nectarine trees, plum trees, and pear trees belong to this group. The third group consists of early-flowering fruit trees (February-March). Almond trees are the fruit trees that flower from February onwards in Aragon. Finally, there are late-flowering fruit trees (April-May), those whose flowering is concentrated mainly in the month of May (45% on average ±21%). Apple trees, quince trees, and other pear varieties make up this group.

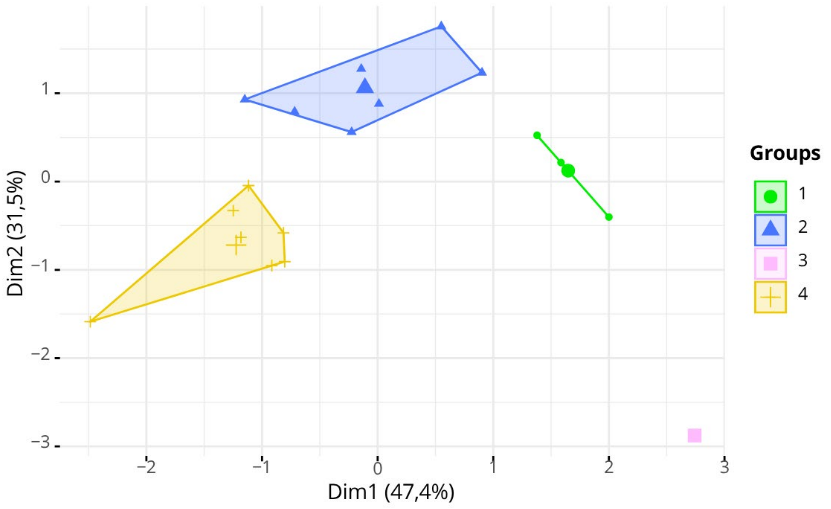

Supplementary Figure S2. Cluster of flowering periods of fruit trees in Spain. Source: Prepared by the authors using data from the Ministerio de Agricultura, Pesca y Alimentación (2024a, 2024b). Dim1 and Dim2 are the two new dimensions obtained by *kmeans* and are used to visualize data clusters in a simplified form. These two dimensions explain more than 80% of the variance in total variability, and in this context, this means that the two-dimensional projection captures most of the differences between crops. The separation of the groups also indicates that the clusters are well-defined and that the flowering patterns are distinct.

D. Spatial distribution of fruit plantations in Aragon

Supplementary Figure S3 shows the location for fruit trees plantation according to SIGPAC, for its acronym in Spanish (Gobierno de Aragón, 2024). Two of the main production regions are located in the west (Jalón) at altitudes above 400 meters above sea level, and in the east, where the plantations are mostly concentrated between 200 and 400 meters above sea level.

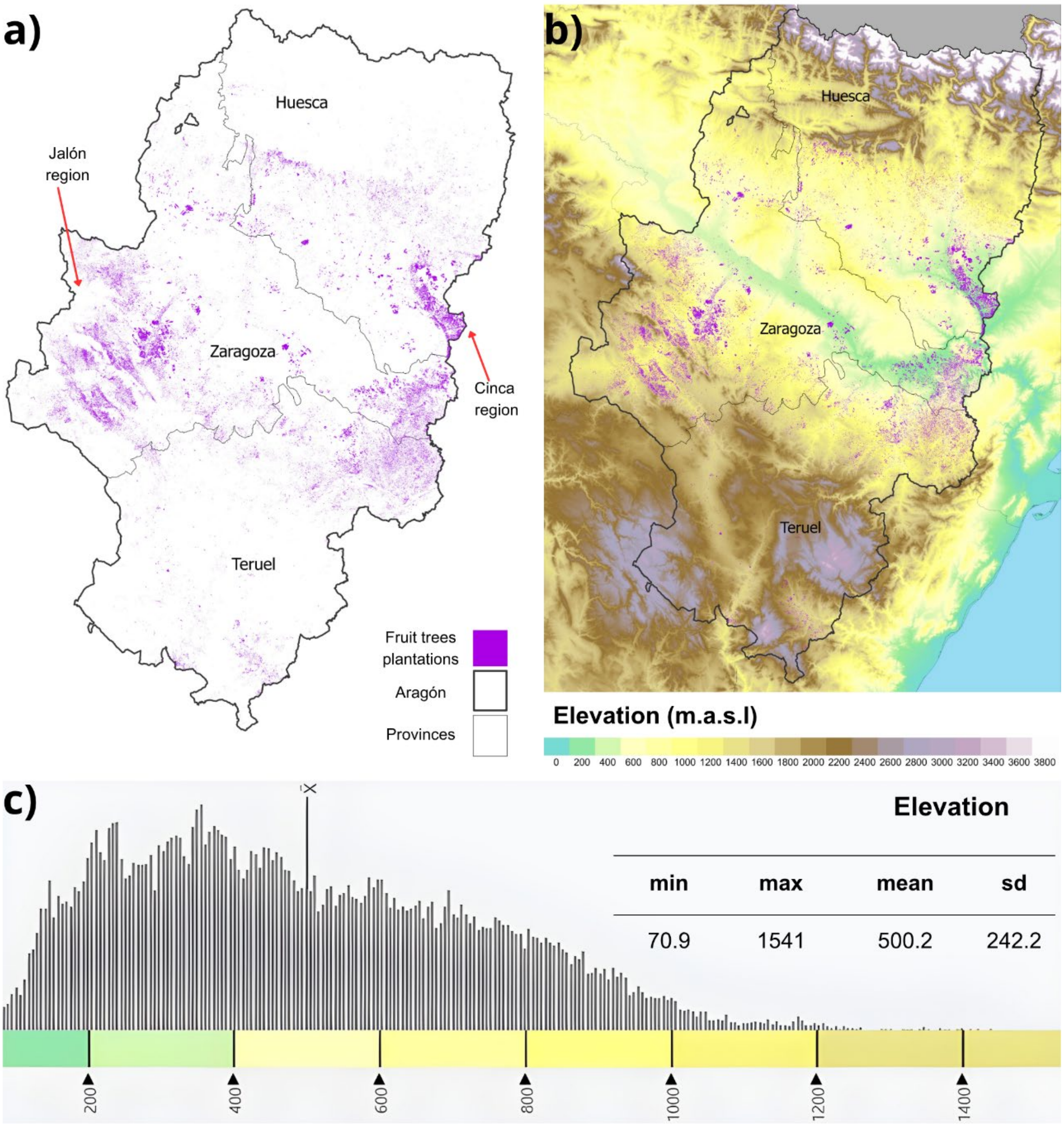

Supplementary Figure S3. Spatial distribution of fruit trees plantations in Aragón and its main production areas (**a**). Altitude in fruit production areas (**b-c**). It is possible to notice that fruit production in Aragon above 1000 meters above sea level is low; the majority of plantations are concentrated between 200 and 800 meters above sea level. Source: Prepared by the authors using data from the Gobierno de Aragón (2024).

E. Anomalies in Aragón

The **Supplementary Tables S5-S9** presents, for each variable, the percentage of observations with positive, negative, and zero changes. Positive changes correspond to values greater than zero, negative changes to values less than zero, and zero changes to values equal to zero. This breakdown allows for assessing the direction and relative magnitude of variations in each variable.

Supplementary Table S5. Statistical summary of CP differences between periods and baseline scenario 1971-2000 Aragón.

CP differences

Scenarios	Mean	Median	SD	No change (%)	Positive (%)	Negative (%)
1951-1980 vs 1971-2000	-0.49	-0.31	1.35	0.00	39.0	61.0
1961-1990 vs 1971-2000	-0.43	-0.29	0.83	0.00	28.8	71.2
1981-2010 vs 1971-2000	-0.17	-0.22	0.50	0.00	25.6	74.4
1991-2020 vs 1971-2000	0.02	-0.05	0.89	0.00	46.0	54.0
2041-2071 (RCP4.5) vs 1971-2000	5.46	5.46	1.57	0.00	99.5	0.5
2071-2100 (RCP4.5) vs 1971-2000	4.98	4.73	2.60	0.00	98.6	1.4
2041-2071 (RCP8.5) vs 1971-2000	4.85	4.44	3.03	0.00	97.4	2.6
2071-2100 (RCP8.5) vs 1971-2000	2.13	1.54	6.41	0.00	60.8	39.2

Supplementary Table S6. Statistical summary of Sa CP between periods and baseline scenario 1971-2000 Aragón.

Sa CP

Scenarios	Mean	Median	SD	No change (%)	Positive (%)	Negative (%)
1951-1980 vs 1971-2000	-0.06	-0.04	0.16	0.00	39.0	61.0
1961-1990 vs 1971-2000	-0.05	-0.04	0.10	0.00	28.8	71.2
1981-2010 vs 1971-2000	-0.02	-0.03	0.06	0.00	25.6	74.4
1991-2020 vs 1971-2000	0.01	-0.01	0.11	0.00	46.0	54.0
2041-2071 (RCP4.5) vs 1971-2000	0.73	0.73	0.20	0.00	99.5	0.5
2071-2100 (RCP4.5) vs 1971-2000	0.66	0.63	0.33	0.00	98.6	1.4
2041-2071 (RCP8.5) vs 1971-2000	0.64	0.59	0.39	0.00	97.4	2.6
2071-2100 (RCP8.5) vs 1971-2000	0.28	0.20	0.81	0.00	60.8	39.2

Supplementary Table S7. Statistical summary of GDH differences between periods and baseline scenario 1971-2000 Aragón.

GDH differences						
Scenarios	Mean	Median	SD	No change (%)	Positive (%)	Negative (%)
1951-1980 vs 1971-2000	-449.2	-431.4	148.0	0.00	0.5	99.5
1961-1990 vs 1971-2000	-372.7	-367.1	120.0	0.00	0.1	99.9
1981-2010 vs 1971-2000	247.5	258.6	124.1	0.00	96.1	3.9
1991-2020 vs 1971-2000	334.2	326.3	187.6	0.00	97.0	3.0
2041-2071 (RCP4.5) vs 1971-2000	517.4	494.5	360.9	0.00	92.4	7.6
2071-2100 (RCP4.5) vs 1971-2000	1049.3	1065.3	476.2	0.00	96.9	3.1
2041-2071 (RCP8.5) vs 1971-2000	1093.2	1113.3	494.7	0.00	96.9	3.1
2071-2100 (RCP8.5) vs 1971-2000	1049.3	1065.3	476.2	0.00	96.9	3.1

Supplementary Table S8. Statistical summary of Sa GDH between periods and baseline scenario 1971-2000 Aragón.

Sa GDH						
Scenarios	Mean	Median	SD	No change (%)	Positive (%)	Negative (%)
1951-1980 vs 1971-2000	-0.36	-0.37	0.11	0.00	0.5	99.5
1961-1990 vs 1971-2000	-0.30	-0.31	0.07	0.00	0.1	99.9
1981-2010 vs 1971-2000	0.21	0.22	0.09	0.00	96.1	3.9
1991-2020 vs 1971-2000	0.28	0.27	0.15	0.00	97.0	3.0
2041-2071 (RCP4.5) vs 1971-2000	0.37	0.38	0.32	0.00	92.4	7.6
2071-2100 (RCP4.5) vs 1971-2000	0.80	0.81	0.37	0.00	96.9	3.1
2041-2071 (RCP8.5) vs 1971-2000	0.83	0.85	0.38	0.00	96.9	3.1
2071-2100 (RCP8.5) vs 1971-2000	0.80	0.81	0.37	0.00	96.9	3.1

Supplementary Table S9. Statistical summary of SFPO (%) differences between periods and baseline scenario 1971-2000 Aragón.

SFPO differences						
Scenarios	Mean	Median	SD	No change (%)	Positive (%)	Negative (%)
1951-1980 vs 1971-2000	95.7	99.0	8.0	62.3	15.6	22.2
1961-1990 vs 1971-2000	95.4	99.0	7.1	62.3	15.6	22.2
1981-2010 vs 1971-2000	95.4	99.0	7.1	67.0	1.5	31.4
1991-2020 vs 1971-2000	94.1	99.0	10.0	62.2	1.2	36.5
2041-2071 (RCP4.5) vs 1971-2000	91.7	99.0	14.3	8.2	0.0	91.8
2071-2100 (RCP4.5) vs 1971-2000	36.0	16.6	34.9	5.6	0.0	94.3
2041-2071 (RCP8.5) vs 1971-2000	30.5	17.6	29.2	4.2	0.0	95.8
2071-2100 (RCP8.5) vs 1971-2000	27.0	13.0	29.8	0.0	0.1	99.9

F. Statistical summary of CP-GDH and CP-SFPO in fruit trees plantations

Supplementary Table S10. Statistical summary of CP-GDH values between periods and baseline scenario 1971-2000 in fruit trees areas in Aragón.

	CP				GDH				
	**q1**	**q2**	**q3**	**IQR**	**q1**	**q2**	**q3**	**IQR**	**rho**
1951-1980	81.3	82.8	83.4	2.1	3979.0	4881.0	5827.0	1847.8	-0.7
1961-1990	80.8	82.4	83.3	2.4	4102.0	4941.0	5871.0	1768.8	-0.8
1971-2010	81.0	82.6	83.3	2.3	4471.0	5350.0	6255.0	1784.2	-0.9
1981-2010	80.7	82.3	83.2	2.5	4766.0	5591.0	6420.0	1653.8	-0.8
1991-2020	80.8	82.3	83.3	2.5	4808.0	5684.0	6539.0	1730.8	-0.8
2041-2070 (RCP4.5)	85.3	87.4	88.8	3.4	5058.0	6007.0	7076.0	2018.3	-0.9
2071-2100 (RCP4.5)	83.3	86.1	87.8	4.4	5619.0	6606.0	7753.0	2133.5	-0.9
2041-2070 (RCP8.5)	82.9	85.7	87.5	4.6	5670.0	6661.0	7820.0	2150.4	-0.9
2071-2100 (RCP8.5)	76.2	81.2	84.1	7.9	5578.0	6551.0	7697.0	2118.3	-0.9

Supplementary Table S11. Statistical summary of CP-GDH standardized anomalies between periods and baseline scenario 1971-2000 in fruit trees areas in Aragón.

	Sa CP				*Sa* GDH				
	**q1**	**q2**	**q3**	**IQR**	**q1**	**q2**	**q3**	**IQR**	**rho**
1951-1980 vs 1971-2000	-0.04	0.01	0.06	0.11	-0.31	-0.27	-0.22	0.08	-0.30
1961-1990 vs 1971-2000	-0.06	-0.01	0.02	0.08	-0.31	-0.27	-0.22	0.08	-0.40
1981-2010 vs 1971-2000	-0.05	-0.03	0.00	0.05	0.13	0.20	0.25	0.12	-0.21
1991-2020 vs 1971-2000	-0.06	-0.01	0.03	0.09	0.16	0.25	0.35	0.19	-0.43
2041-2071 (RCP4.5) vs 1971-2000	0.54	0.65	0.74	0.20	0.34	0.44	0.60	0.24	-0.59
2071-2100 (RCP4.5) vs 1971-2000	0.29	0.49	0.61	0.31	0.78	0.90	1.06	0.27	-0.58
2041-2071 (RCP8.5) vs 1971-2000	0.23	0.42	0.55	0.31	0.83	0.95	1.12	0.28	-0.58
2071-2100 (RCP8.5) vs 1971-2000	-0.68	-0.17	0.10	0.78	0.78	0.89	1.06	0.27	-0.58

Supplementary Table S12. Statistical summary of CP-SFPO values between periods and baseline scenario 1971-2000 in fruit trees areas in Aragón.

	CP				SFPO (%)				
	**q1**	**q2**	**q3**	**IQR**	**q1**	**q2**	**q3**	**IQR**	**rho**
1951-1980	81.3	82.8	83.4	2.1	90.3	96.7	99.9	9.6	0.7
1961-1990	80.8	82.4	83.3	2.4	90.3	96.7	99.9	9.6	0.8
1971-2010	81.0	82.6	83.3	2.3	90.3	96.7	99.9	9.6	0.7
1981-2010	80.7	82.3	83.2	2.5	87.1	93.5	99.9	12.9	0.7
1991-2020	80.8	82.3	83.3	2.5	80.6	93.5	99.9	19.3	0.6
2041-2070 (RCP4.5)	85.3	87.4	88.8	3.4	7.6	10.3	16.1	8.0	0.6
2071-2100 (RCP4.5)	83.3	86.1	87.8	4.4	4.1	10.7	17.8	13.6	0.6
2041-2070 (RCP8.5)	82.9	85.7	87.5	4.6	5.0	8.0	12.0	7.0	0
2071-2100 (RCP8.5)	76.2	81.2	84.1	7.9	NA	NA	NA	NA	NA

Supplementary Table S13. Statistical summary of CP- SFPO differences between periods and baseline scenario 1971-2000 in fruit trees areas in Aragón.

	Δ CP				Δ SFPO (%)				
	**q1**	**q2**	**q3**	**IQR**	**q1**	**q2**	**q3**	**IQR**	**rho**
1951-1980 vs 1971-2000	-0.38	0.08	0.51	0.90	-3.22	0.00	0.86	4.08	-0.11
1961-1990 vs 1971-2000	-0.48	0.16	0.17	0.65	-3.22	0.00	0.53	3.76	-0.10
1981-2010 vs 1971-2000	-0.44	-0.23	-0.03	0.41	-3.22	0.00	0.00	3.22	0.17
1991-2020 vs 1971-2000	-0.05	-0.12	0.21	0.71	-9.67	-3.11	0.00	9.67	-0.10
2041-2071 (RCP4.5) vs 1971-2000	4.18	4.95	5.51	1.33	-86.6	-80.06	-73.44	13.22	0.26
2071-2100 (RCP4.5) vs 1971-2000	2.48	3.72	4.53	2.05	-87.10	-80.65	-70.97	16.12	0.31
2041-2071 (RCP8.5) vs 1971-2000	1.85	3.17	4.02	2.17	-90.0	-84.64	-76.67	13.33	-0.25
2071-2100 (RCP8.5) vs 1971-2000	-4.88	-1.25	0.76	5.65	NA	NA	NA	NA	NA

G. Statistical summary of CP, GDH and SFPO magnitude, direction and variability in east and west fruit trees areas in Aragon

Magnitude (mean absolute anomaly) represents the overall intensity of change, ignoring sign, where high values are interpreted as strong changes (either positive or negative). Direction (mean anomaly) indicates the predominant trend of change, where positive values are associated with an increasing average change, and negative values with a decreasing average change. Finally, Variability (sd of anomalies) relates to the dispersion of changes over time, where high values can be interpreted as heterogeneous and inconsistent responses over time, and low values as consistent and stable changes.

Supplementary Table S14. Statistical summary of magnitude, direction and variability of CP in both regions.

			**CP Magnitude**			
	Mean	q2	sd	q1	q3	IQR
East	0.26	0.25	0.04	0.23	0.28	0.04
West	0.30	0.29	0.06	0.25	0.33	0.08
			**CP Direction**			
	Mean	q2	sd	q1	q3	IQR
East	0.02	0.02	0.14	-0.08	0.14	0.22
West	0.24	0.24	0.08	0.19	0.30	0.11
			**CP Variability**			
	Mean	q2	sd	q1	q3	IQR
East	0.39	0.38	0.08	0.32	0.45	0.12
West	0.35	0.34	0.49	0.31	0.37	0.06

Supplementary Table S15. Statistical summary of magnitude, direction and variability of GDH in both regions.

			**GDH Magnitude**			
	Mean	q2	sd	q1	q3	IQR
East	0.59	0.57	0.14	0.48	0.70	0.22
West	0.49	0.49	0.08	0.44	0.54	0.10
			**GDH Direction**			
	Mean	q2	sd	q1	q3	IQR
East	0.46	0.44	0.14	0.36	0.57	0.21
West	0.34	0.34	0.09	0.28	0.40	0.11
			**GDH Variability**			
	Mean	q2	sd	q1	q3	IQR
East	0.58	0.57	0.11	0.50	0.66	0.16
West	0.48	0.46	0.07	0.44	0.51	0.07

Supplementary Table S16. Statistical summary of magnitude, direction and variability of SFPO in both regions.

			**SFPO Magnitude**			
	Mean	q2	sd	q1	q3	IQR
East	0.42	0.44	0.06	0.38	0.47	0.08
West	0.43	0.44	0.04	0.41	0.45	0.04
			**SFPO Direction**			
	Mean	q2	sd	q1	q3	IQR
East	-0.41	-0.43	0.07	-0.47	-0.36	0.10
West	-0.43	-0.43	0.04	-0.45	-0.43	0.05
			**SFPO Variability**			
	Mean	q2	sd	q1	q3	IQR
East	0.40	0.42	0.07	0.35	0.47	0.12
West	0.45	0.46	0.03	0.43	0.48	0.04
